# Change in Work-Family Spillover across 20 Years: Longitudinal Analysis of the Midlife in the United States Study

**DOI:** 10.1093/geroni/igaf122.3009

**Published:** 2025-12-31

**Authors:** Jeongmin Park, Kyoungmin Cho, Soomi Lee, David Almeida

**Affiliations:** The Pennsylvania State University, University Park, Pennsylvania, United States; The Pennsylvania State University, University Park, Pennsylvania, United States; The Pennsylvania State University, University Park, Pennsylvania, United States; The Pennsylvania State University, University Park, Pennsylvania, United States

## Abstract

**Background:**

Work and family are two of the most central roles in adulthood, yet little is known about how these experiences evolve with aging. This study examined changes in work–family spillover over a 20-year period and explored whether long-term trajectories vary by age and gender.

**Methods:**

Data were from the Midlife in the United States (MIDUS) study across three waves (N = 665, employed at all waves; 1995-1996, 2004-2006, 2013-2014). Four types of work–family spillover were measured: negative work-to-family, negative family-to-work, positive work-to-family, and positive family-to-work. Multilevel growth curve models with random slopes and intercepts assessed longitudinal trajectories of work–family spillover controlling for baseline age, gender, race, and education. We also tested whether changes in work-family spillover differed by age and gender.

**Results:**

Negative spillover (work-to-family and family-to-work) declined over time, with no quadratic effects. Positive spillovers followed an inverted U-shape, increasing from the first to the second wave and declining by the third. Older adults experienced steeper declines in negative spillovers, but their changes in positive spillovers were similar with those of younger adults. Gender was not associated with changes over time, although females reported higher initial levels of negative family-to-work spillover. These patterns remained consistent after covariate adjustment.

**Conclusions:**

Work-family spillover change over time, with negative spillover showing a linear decline and positive spillovers following an increase-decrease pattern. Age-related differences in negative spillover trajectories and gender differences in initial negative spillover levels highlight the need for workplace policies that support work-family balance for different population groups.

